# CONCERN: Does ovary need D-chiro-inositol?

**DOI:** 10.1186/1757-2215-5-14

**Published:** 2012-05-15

**Authors:** Rosalbino Isabella, Emanuela Raffone

**Affiliations:** 1 C.I.S. Reproductive Medicine, Lamezia Terme, Italy; 2 Obstetrics and Gynecology Department, G. Martino Hospital, Messina, Italy

**Keywords:** Metformin, PCOS Patient, Ovarian Response, PCOS Woman, Oocyte Quality

## Abstract

**Backgroud:**

Polycystic Ovary Syndrome (PCOS) is a multifactorial pathology that affects 10% of the women in reproductive age being the main cause of infertility due to menstrual dysfunction. Since 1980, it is known that PCOS is associated with insulin resistance (IR). The recognition of this association has prompted extensive investigation on the relationship between insulin and gonadal function, and has turned insulin sensitizer agent as the main therapeutic choice. In particular two different polyalcohol myo-inositol and D-chiro-inositol have been shown to improve insulin resistance, hyperandrogenism and to induce ovulation in PCOS women. In particular, while data on myo-inositol and restored ovulation were consistent, data on D-chiro-inositol were not . Recently, a comparative study, proposed a D-chiro-inositol paradox in the ovary of PCOS patients hypothesizing that only myo-inositol has a specific ovarian action. In the present study we aim to further study the role played by D-chiro-inositol at ovarian level.

**Methods:**

A total of 54 women, aged <40 years and diagnosed with PCOS were enrolled in this study. Patients with insulin resistance and/or hyperglycaemia were excluded from the study. Patients were randomly divided into 5 groups (n=10-12): a placebo group, and 4 groups (A-D) that received 300-600-1200-2400 mg of DCI daily respectively. All treatments were carried out for 8 weeks before follicle stimulating hormone (rFSH) administration.

**Results:**

Total r-FSH units increased significantly in the two groups that received the higher doses of DCI. The number of immature oocytes was significantly increased in the three groups that received the higher doses of DCI. Concurrently, the number of MII oocytes was significantly lower in the D group compared to placebo group. Noteworthy, the number of grade I embryos was significantly reduced by DCI supplementation.

**Conclusions:**

Indeed, increasing DCI dosage progressively worsens oocyte quality and ovarian response.

**Electronic supplementary material:**

The online version of this article (doi:10.1186/1757-2215-5-14) contains supplementary material, which is available to authorized users.

## Introduction

Polycystic Ovary Syndrome (PCOS) is a multifactorial pathology that affects 10% of the women in reproductive age being the main cause of infertility due to menstrual dysfunction[1–3]. Diagnosis is based on three different factors, identified during a consensus meeting sponsored by the European Society for Human Reproduction and Embryology (ESHRE) and the American Society for Reproductive Medicine (ASRM) held in Rotterdam in 2003: (1) oligo-anovulation, (2) hyperandrogenism (clinical or biochemical) and (3) the presence of 12 or more follicles in each ovary measuring 2–9 mm in diameter, and/or an increased ovarian volume (>10 ml)[2, 3]. The identification of two out of three parameters allows to give the diagnosis of PCOS.

Since 1980, it is known that PCOS is associated with insulin resistance (IR). The recognition of this association has prompted extensive investigation on the relationship between insulin and gonadal function [4]. Insulin acts synergistically with luteinizing hormone (LH) to enhance the androgen production of theca cells [5]. Furthermore, it is able to reduce circulating levels of sex hormone binding globulin (SHBG), leading to increased levels of free testosterone [6]. It has become clear that this syndrome has major metabolic as well as reproductive morbidities. Although the consensus meeting has decided to exclude IR from the diagnostic criteria, IR affects 50-80% of the patients with PCOS regardless BMI[7]. Accordingly, this association has led to the treatment of PCOS women with insulin sensitizing agents such as troglitazone [8], inositol [9, 10], metformin [11] for restoring spontaneous ovulation.

In particular, several lines of evidence suggest that a deficiency of inositol, which is a second messenger of the insulin signaling, may be linked to insulin resistance[12]. Inositol is a polyalcohol classified as insulin sensitizer and existing as nine stereoisomers, two of which are currently used in PCOS treatment: myo-inositol (MI) and D-*chiro*-inositol (DCI) [9, 13]. Both stereoisomers show an insulin-like action *in vivo* exerting the function of insulin mediators as inositolphosphoglycans (IPGs) [14]. Indeed, MI is the most abundant form of inositol in humans while DCI is synthetized by an insulin-dependent epimerase that converts MI to DCI. Interestingly, every organ has a specific MI/DCI ratio likely linked to its specific needs (i.e. specific biological processes controlled be each inositol). Indeed, beside the common features, both inositols have specific action. DCI is able to induce glycogen synthesis; in particular, high DCI levels were identified only in glycogen storage tissues[14]. On the other hand, MI plays a crucial role at ovarian level. Recently, Chiu et al. have reported that high concentrations of MI positively correlate with high quality and mature oocytes and a recent review clearly summarized the important role of MI in human reproduction [15][16]. Furthermore, MI supplementation during IVF protocols has been shown to improve oocyte quality and reduce the number of IU of FSH necessary for ovarian stimulation [17, 18].

Oocyte quality is the main factor influencing the chance of a pregnancy; indeed, poor oocyte quality is a cause of infertility as well as an important obstacle for a successful *in vitro* fertilization (IVF).

Additional data have also shown that DCI supplementation at the dose of 1,2 g/die has no influence on ovarian response to exogenous FSH [19]. The aim of the present study was to better investigate whether DCI has a role at ovarian level.

## Materials and methods

### Patients

A total of 54 women, aged <40 years and diagnosed with PCOS according to Rotterdam criteria, were enrolled in this study. All the subjects were selected among patients undergoing ICSI procedure that was suggested after evaluation of two different sperm samples of the male partner. ICSI patients were selected in order to minimize differences between semen samples. Patients with insulin resistance and/or hyperglycaemia were excluded from the study. In particular, insulin resistance was assessed by calculating the HOMA index, the cut-off defining insulin resistance was 3.2. Concomitantly hyperglycaemia was defied as glucose level > than 140 mg/dl after 2h OGTT Patients were randomly divided into 5 groups (n = 10-12): a placebo group, and 4 groups (A-D) that received 300-600-1200-2400 mg of DCI (Interquim, s.a., Barcelona, Spain) daily respectively. All treatments were carried out for 8 weeks before follicle stimulating hormone (rFSH) administration. The randomization procedure was performed using a computer-based program. The Institutional Ethical Committee approved the protocol, and all patients gave a written informed consent before entering the study.

### Controlled ovarian hyperstimulation

All patients underwent pituitary desensitization by subcutaneous (s.c.) administration of the gonadotropin releasing hormone (GnRH) agonist (Decapeptyl; Ipsen, Paris, France) from midluteal phase until the day of intramuscular (i.m.) administration of 10,000 IU of human chorionic gonadotropin (hCG). Then, controlled ovarian hyperstimulation was performed in all patients by the administration of recombinant FSH (Gonal-F; Merck-Serono, Geneva, Switzerland). Starting dose was 150 IU per day. Patients were monitored by measuring the plasma concentration of 17β-Estradiol 2 (17β-E_2_) and the size of follicles on day 5 of the stimulation. The dosage of gonadotropin was adjusted according to the individual response. The 10,000 IU hCG was injected i.m. in all patients when serum 17β-E_2_ exceeded 200 pg per follicle and at least three follicles with a minimum diameter of 18 mm were found. Cycles were cancelled if E_2_ levels were >4,000 pg/mL, due to increased risk of ovarian hyperstimulation syndrome (OHSS).

### ICSI procedure

According to Italian IVF law, a maximum of three oocytes per patient were injected, while spare mature oocytes were cryopreserved according to protocols described in previous studies [20]. Oocyte and sperm preparation for conventional ICSI procedure have been thoroughly described elsewhere [21]. Concerning ICSI, cumulus and corona radiata cells were immediately removed after retrieval by a short exposure to HEPES-buffered medium (Quinn’s Advantage Hepes Medium; Sage IVF, Trumbull, CT, USA) containing 20 IU/mL hyaluronidase (Sage IVF) and by gentle aspiration in and out of a Pasteur pipette following by mechanical cleaning from the remaining surrounding cumulus cells by aspiration using a denuding pipette (Denuding Flexi-Pet; Cook, Brisbane, Australia) with a 170–130 mm diameter. The denuded oocytes were then assessed for their meiotic maturation status. In preparation for ICSI, oocytes with an extruded first polar body presumably at the metaphase II stage (MII) were selected (in a maximum of three) for the fresh cycle and spare MII oocytes were cryopreserved, if required [22].

### Luteal phase

Intramuscular administration of 50 mg daily progesterone in-oil was started on the day of ovum pick-up, and the treatment was performed daily until either a serum pregnancy test result was negative or an embryonic heart beat was sonographically confirmed.

### Statistical analysis

Baseline characteristics and ovum pick-up outcomes were analyzed using One-way ANOVAs. The source of the detected significances was determined by Bonferroni post hoc test. *P* values less than 0.05 were considered statistically significant.

## Results

**Figure 1:**
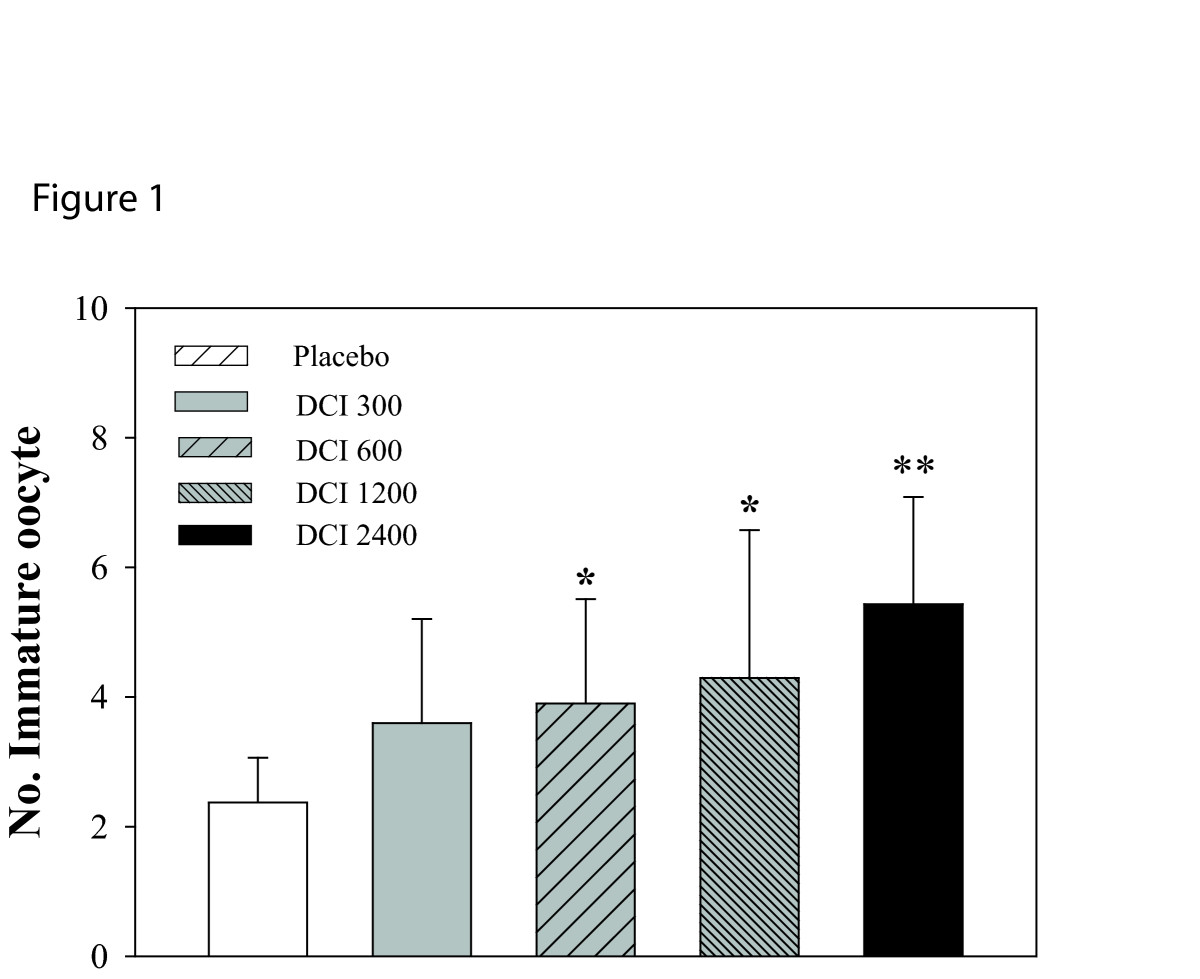
**Effects of DCI (300, 600, 2400 mg) on number of immature oocytes.** Data are expressed as mean ± SD. Number of immature oocytes increases after DCI administration. *, P <0.05 vs. placebo;**, P <0.001 vs. placebo (n = 10-11 per group)

**Figure 2:**
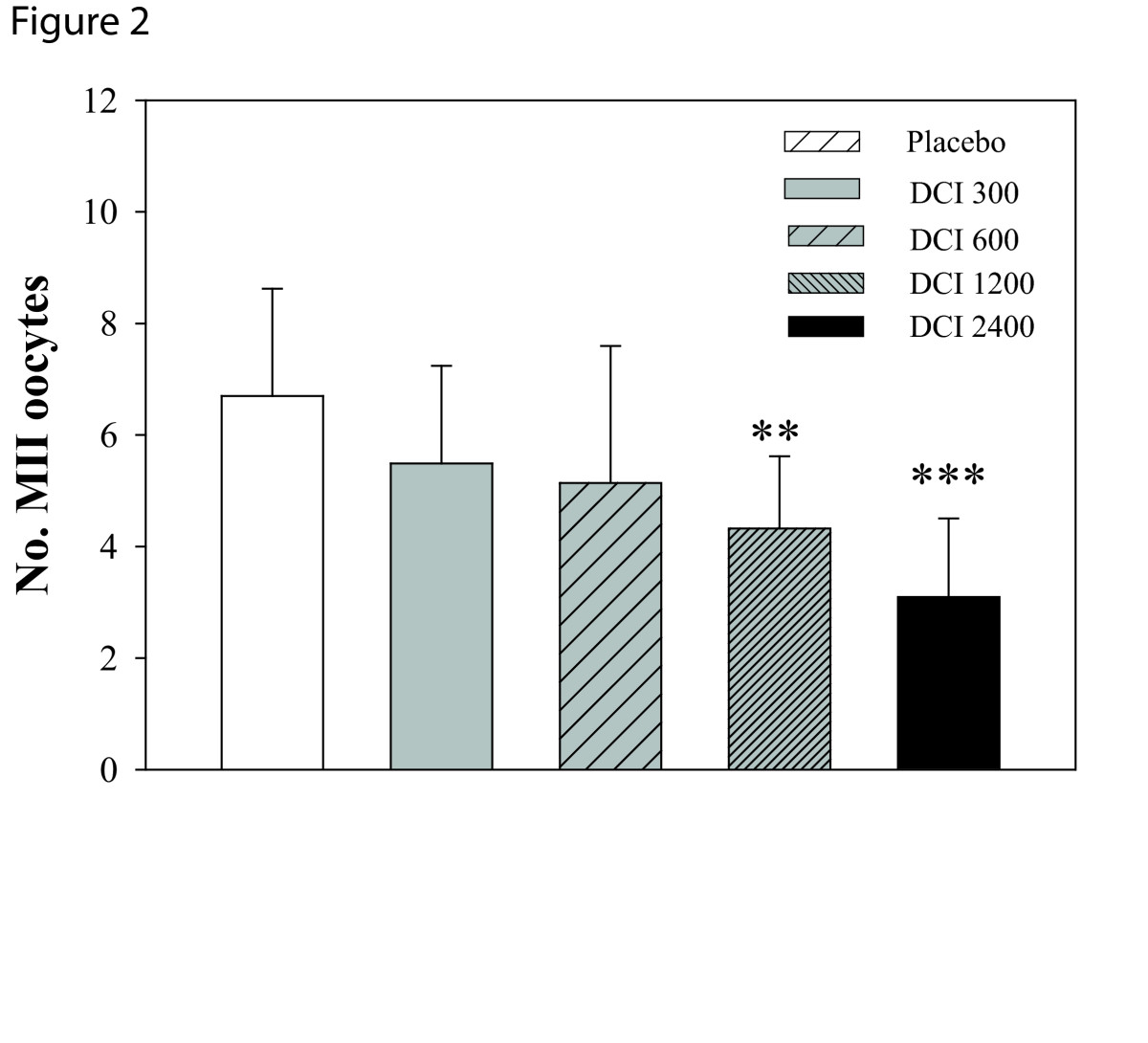
**Effects of DCI (300, 600, 2400 mg) on number of MII oocytes.** Data are expressed as mean ± SD. Number of MII occytes decreases after DCI administration. **P <0.01 vs.placebo, ***, P <0.001 vs.placebo (n = 10-11 per group)

**Figure 3:**
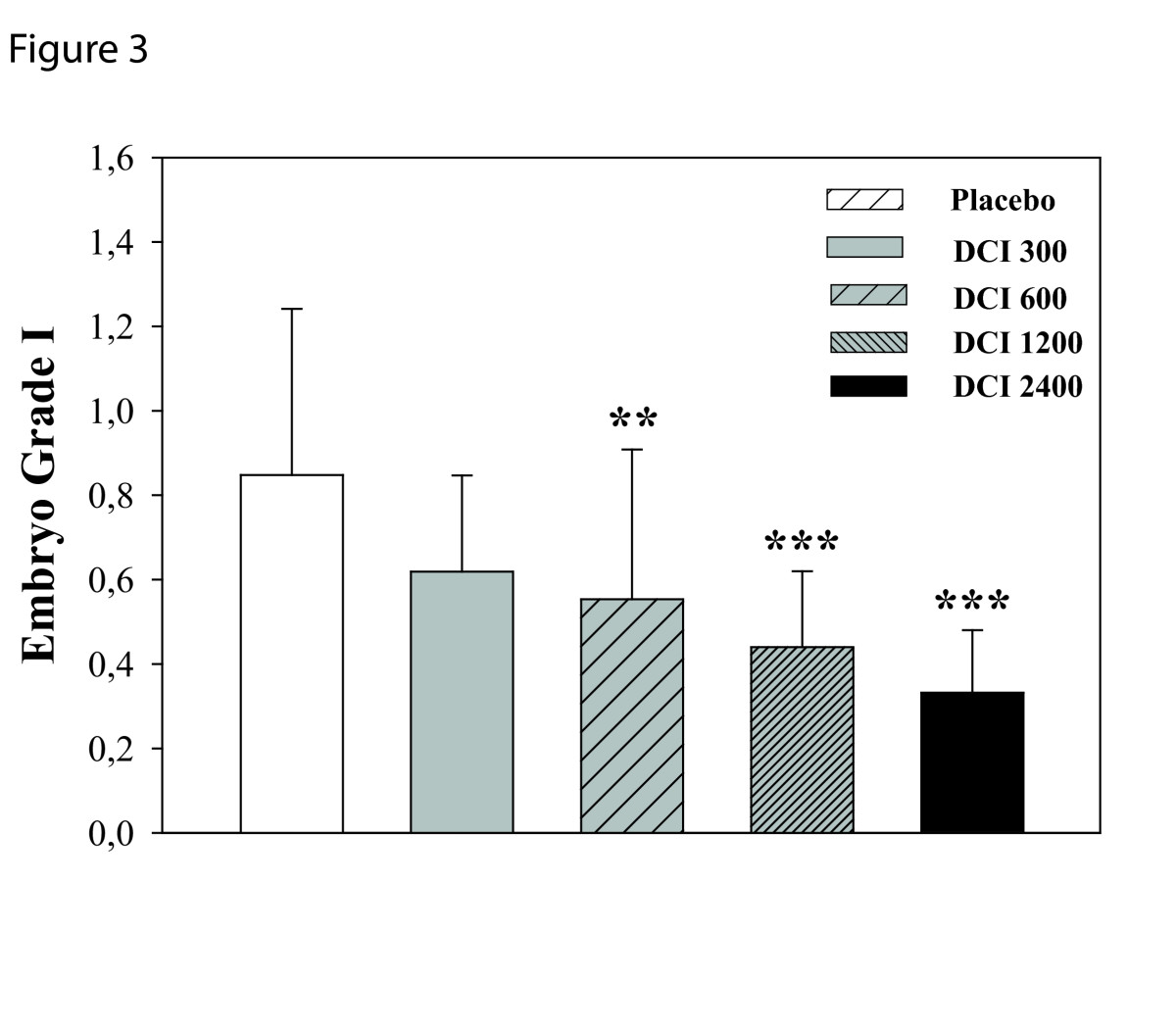
**Effects of DCI (300, 600, 2400 mg) on number of embryo grade I.** Data are expressed as mean ± SD. Number of embryo grade I decreases after DCI administration. **, P <0.01 vs. placebo, ***, P <0.001 vs. placebo (n = 10-11 per group)

Patients matching the inclusion criteria were randomized into five groups by a computer program. One-way ANOVA revealed no significant differences between groups in mean age, Body Mass Index at baseline (Table 1). Total r-FSH units and number of days of stimulation differed between the groups. In particular, compared to the placebo group, the FSH IU administered were increased significantly in the two groups that received the higher doses of DCI (Table 2). Estradiol levels at hCG administration were significantly different only in the group D (women treated with 1.2g of DCI Table 2). The total number of oocytes retrieved were similar between the groups, while the number of immature oocytes (Figure 1, P < 0.003) and number of MII oocytes (Figure 2, P = 0.0013) significantly differed. Indeed, the number of immature oocytes was significantly increased in the three groups that received the higher doses of DCI (P < 0.04). Concurrently, the number of MII oocytes was significantly lower in the D group compared to placebo group (P < 0.001). Noteworthy, the number of grade I embryos was significantly reduced by DCI supplementation (P = 0.004). Embryo quality was significantly reduced in women treated with 600, 1200 and 2400 mg of DCI (Figure 3).

**Table 1:** **Baseline characteristics of patients**

	PLACEBO	A (DCI 300mg)	B (DCI 600mg)	C (DCI 1200mg)	D (DCI 2400mg)	***p***
No. of patients	11	10	11	10	12	NS
Age (years)	36.9 ± 1.5	36.8 ± 1.6	36.9 ± 1.52	36.7 ± 1.57	37.0 ± 1.25	NS
BMI (kg/m^2^)	24.4 ± 2.8	25.2 ± 3.5	24.7 ± 3.5	25.1 ± 3.1	25.6 ± 2.9	NS
Duration of infertility (months)	48.2 ± 9.4	49.4 ± 7.6	50.0 ± 7.2	49.9 ± 6.1	48.9 ± 8.8	NS

Data are expressed as mean ± SD.

**Table 2:** **Clinical Data**

	PLACEBO	A (DCI 300 mg)	B (DCI 600mg)	C (DCI 1200mg)	D (DCI 2400mg)
No. Oocytes retrieved	8.99 ± 2.52	9.20 ± 2.46	9.13 ± 2.99	7.83 ± 2.78	7.23 ± 2.77
Total rFSH (IU)	2239.7 ± 181.55	2379.1 ± 353.80	2305.9 ± 150.19	2368.5 ± 235.77*	2983.0 ± 219.80**
17β-E2 levels (pg/ml) on hGC administration	1429.69 ± 1118.43	1443.43 ± 1087.43	1350.06 ± 513.04	1530.85 ± 433.17	1490.24 ± 253.21 *
Stimulation (days)	11.4 ± 1.2	12.1 ± 0.99	12.5 ± 1.21 *	12.9 ± 1.10 **	13.8 ± 0.87**
No. Of cycles cancelled	1	1	0	0	2

Data are expressed as mean ± SD.

17β-E2 levels decreases in D group.

*, P < 0.05 vs placebo. Stimulation days increase in B,C,D group (300, 600, 1200 mg DCI, respectively).

*, P < 0.05 vs placebo, **, P < 0.01 vs placebo.

## Discussion

In the present study we were able to show that DCI negatively affects oocyte quality. Indeed, increasing DCI dosage progressively worsens oocyte quality and ovarian response in non-obese and non insulin resistant PCOS women. Literature data clearly show that the two inositol stereoisomers have different physiological roles: while DCI is crucial for glycogen synthesis, MI increases glucose cellular uptake [12]. Interestingly, each tissue has its own MI/DCI ratio and the relative amount of each inositol in a certain tissue reflects inositol(s) specific functions. Indeed, high DCI levels (always lower than MI) are present only in glycogen storage tissues (fat, liver and muscle), while very low levels of DCI are characteristic of tissues that have high glucose utilization, likely because they need to “have a high energy status” (brain and heart) [14]. Recently a new manuscript pointed out that one of the causes of poor oocyte quality in PCOS women might be the reduced energy metabolism [23]. Indeed, literature data have shown that in PCOS women, genes involved in the glucose uptake pathway are downregulated at ovarian level [24]. These data are indeed in line with the findings of Carlomagno et al., that in a comparative study (MI vs. DCI) highlighted that only MI has an action at ovarian level; since MI is responsible of glucose cell uptake, likely it improves ovary energy status and therefore, it is able to improve oocyte quality [19, 23]. *In vivo*, DCI is synthetized by an epimerase that converts MI into DCI and, depending on the specific needs of the two different molecules, each tissue has a typical conversion rate. In particular, it has been shown that the ratio of these two insulin mediators is insulin dependent[25, 26]. Indeed, in subjects with type 2 diabetes, the DCI/MI ratio is reduced and less DCI is synthetized, due to a reduction in the epimerase activity [25, 26]. Unlike other tissues such as muscle and liver, ovary never become insulin resistant [27, 28]. Therefore, it can be speculated that PCOS patients with hyperinsulinemia probably present an enhanced MI to DCI epimerization rate in the ovary that would result in an increased DCI/MI ratio (i.e., overproduction of DCI), and in a MI deficiency. This MI depletion could eventually be responsible for the poor oocyte quality observed in these patients [29]. In addition, the positive effects of MI supplementation [17–19, 30] might be explained by the fact that restoring MI levels will improve ovarian response to FSH; furthermore, since MI is responsible for glucose cell intake, energy status will be restored as well. Nestler et al. in 1999 showed for the first time that administration of DCI (1.2 g/d) in obese PCOS women is able to restore ovulation after 4 weeks of treatment, although no data on menstrual regularity were reported[9]. However, in a second study conducted by the same authors, DCI dosage was doubled (2400 mg/die) and authors were not able to confirm their pervious findings.[31] Our results seems to be in line with the DCI paradox hypothesis [29]. Indeed, increasing DCI dosage progressively negatively influences oocyte and embryo quality. Similar results have been obtained by treating PCOS woman scheduled for IVF with metformin, 500 mg three times per day[32]. Metformin decreased the number of dominant follicles, retrieved oocytes, and MII oocytes, despite increased IU of gonadotropin were administered. Baillargoen et al. showed that metformin increased the insulin-stimulated release of DCI-phosphoglycans; therefore, we could hypothesize that metformin promotes glycogen synthesis and further reduces ovary energy status through DCI phosphoglycans [33]. Based on this evidence, we can speculate on the reason why Nesteler had opposite results in the two trials. In the first study, DCI supplementation restored ovulation probably due to a peripheral action of DCI, allowing to restore epimerase physiological rating into the ovary. In the second trial, the lack of positive effects after 2400mg DCI supplementation was probably due to an overload of DCI (pre-existent high levels of DCI in the ovary and supplementation). In conclusion, we have shown that DCI supplementation reduces oocyte quality; therefore the administration of DCI cannot be considered as an appropriate approach to improve IVF outcomes in PCOS patients.

## Authors’ original submitted files for images

Below are the links to the authors’ original submitted files for images. Authors’ original file for figure 1
Authors’ original file for figure 2
Authors’ original file for figure 3

## References

[1] Taylor AE (1998). Polycystic ovary syndrome. Endocrinol Metab Clin North Am.

[2] **Revised 2003 consensus on diagnostic criteria and long-term health risks related to polycystic ovary syndrome** *Fertil Steril* 2004, **81:** 19–25. 10.1016/j.fertnstert.2003.10.004 14711538

[3] **Revised 2003 consensus on diagnostic criteria and long-term health risks related to polycystic ovary syndrome (PCOS)** *Hum Reprod* 2004, **19:** 41–47. 10.1093/humrep/deh098 14688154

[4] Burghen GA, Givens JR, Kitabchi AE (1980). Correlation of hyperandrogenism with hyperinsulinism in polycystic ovarian disease. J Clin Endocrinol Metab.

[5] Baillargeon JP, Carpentier A (2007). Role of insulin in the hyperandrogenemia of lean women with polycystic ovary syndrome and normal insulin sensitivity. Fertil Steril.

[6] Baptiste CG, Battista MC, Trottier A, Baillargeon JP (2010). Insulin and hyperandrogenism in women with polycystic ovary syndrome. J Steroid Biochem Mol Biol.

[7] Ehrmann DA (1999). Insulin-lowering therapeutic modalities for polycystic ovary syndrome. Endocrinol Metab Clin North Am.

[8] Hasegawa I, Murakawa H, Suzuki M, Yamamoto Y, Kurabayashi T, Tanaka K (1999). Effect of troglitazone on endocrine and ovulatory performance in women with insulin resistance-related polycystic ovary syndrome. Fertil Steril.

[9] Nestler JE, Jakubowicz DJ, Reamer P, Gunn RD, Allan G (1999). Ovulatory and metabolic effects of D-chiro-inositol in the polycystic ovary syndrome. N Engl J Med.

[10] Gerli S, Mignosa M, Di Renzo GC (2003). Effects of inositol on ovarian function and metabolic factors in women with PCOS: a randomized double blind placebo-controlled trial. Eur Rev Med Pharmacol Sci.

[11] Ng EH, Wat NM, Ho PC (2001). Effects of metformin on ovulation rate, hormonal and metabolic profiles in women with clomiphene-resistant polycystic ovaries: a randomized, double-blinded placebo-controlled trial. Hum Reprod.

[12] Huang LC, Fonteles MC, Houston DB, Zhang C, Larner J (1993). Chiroinositol deficiency and insulin resistance. III. Acute glycogenic and hypoglycemic effects of two inositol phosphoglycan insulin mediators in normal and streptozotocin-diabetic rats in vivo. Endocrinology.

[13] Papaleo E, Unfer V, Baillargeon JP, De Santis L, Fusi F, Brigante C, Marelli G, Cino I, Redaelli A, Ferrari A (2007). Myo-inositol in patients with polycystic ovary syndrome: a novel method for ovulation induction. Gynecol Endocrinol.

[14] Larner J (2002). D-chiro-inositol–its functional role in insulin action and its deficit in insulin resistance. Int J Exp Diabetes Res.

[15] Chiu TT, Rogers MS, Law EL, Briton-Jones CM, Cheung LP, Haines CJ (2002). Follicular fluid and serum concentrations of myo-inositol in patients undergoing IVF: relationship with oocyte quality. Hum Reprod.

[16] Papaleo E, Unfer V, Baillargeon JP, Chiu TT (2009). Contribution of myo-inositol to reproduction. Eur J Obstet Gynecol Reprod Biol.

[17] Papaleo E, Unfer V, Baillargeon JP, Fusi F, Occhi F, De Santis L (2009). Myo-inositol may improve oocyte quality in intracytoplasmic sperm injection cycles. A prospective, controlled, randomized trial. Fertil Steril.

[18] Raffone E, Rizzo P, Benedetto V (2010). Insulin sensitiser agents alone and in co-treatment with r-FSH for ovulation induction in PCOS women. Gynecol Endocrinol.

[19] Unfer V, Carlomagno G, Rizzo P, Raffone E, Roseff S (2011). Myo-inositol rather than D-chiro-inositol is able to improve oocyte quality in intracytoplasmic sperm injection cycles. A prospective, controlled, randomized trial. Eur Rev Med Pharmacol Sci.

[20] Borini A, Sciajno R, Bianchi V, Sereni E, Flamigni C, Coticchio G (2006). Clinical outcome of oocyte cryopreservation after slow cooling with a protocol utilizing a high sucrose concentration. Hum Reprod.

[21] Van de Velde H, Nagy ZP, Joris H, De Vos A, Van Steirteghem AC (1997). Effects of different hyaluronidase concentrations and mechanical procedures for cumulus cell removal on the outcome of intracytoplasmic sperm injection. Hum Reprod.

[22] De Santis L, Cino I, Rabellotti E, Papaleo E, Calzi F, Fusi FM, Brigante C, Ferrari A (2007). Oocyte cryopreservation: clinical outcome of slow-cooling protocols differing in sucrose concentration. Reprod Biomed Online.

[23] Arya BK, Haq AU, Chaudhury K (2012). Oocyte quality reflected by follicular fluid analysis in poly cystic ovary syndrome (PCOS): A hypothesis based on intermediates of energy metabolism. Med Hypotheses.

[24] Ma X, Fan L, Meng Y, Hou Z, Mao YD, Wang W, Ding W, Liu JY (2007). Proteomic analysis of human ovaries from normal and polycystic ovarian syndrome. Mol Hum Reprod.

[25] Larner J, Craig JW (1996). Urinary myo-inositol-to-chiro-inositol ratios and insulin resistance. Diabetes Care.

[26] Asplin I, Galasko G, Larner J (1993). chiro-inositol deficiency and insulin resistance: a comparison of the chiro-inositol- and the myo-inositol-containing insulin mediators isolated from urine, hemodialysate, and muscle of control and type II diabetic subjects. Proc Natl Acad Sci U S A.

[27] Harwood K, Vuguin P, DiMartino-Nardi J (2007). Current approaches to the diagnosis and treatment of polycystic ovarian syndrome in youth. Horm Res.

[28] Matalliotakis I, Kourtis A, Koukoura O, Panidis D (2006). Polycystic ovary syndrome: etiology and pathogenesis. Arch Gynecol Obstet.

[29] Carlomagno G, Unfer V, Roseff S (2011). The D-chiro-inositol paradox in the ovary. Fertil Steril.

[30] Ciotta L, Stracquadanio M, Pagano I, Carbonaro A, Palumbo M, Gulino F (2011). Effects of myo-inositol supplementation on oocyte's quality in PCOS patients: a double blind trial. Eur Rev Med Pharmacol Sci.

[31] Cheang KI, Baillargeon JP, Essah PA, Ostlund RE, Apridonize T, Islam L, Nestler JE (2008). Insulin-stimulated release of D-chiro-inositol-containing inositolphosphoglycan mediator correlates with insulin sensitivity in women with polycystic ovary syndrome. Metabolism.

[32] Palomba S, Falbo A, Di Cello A, Cappiello F, Tolino A, Zullo F (2011). Does metformin affect the ovarian response to gonadotropins for in vitro fertilization treatment in patients with polycystic ovary syndrome and reduced ovarian reserve? A randomized controlled trial. Fertil Steril.

[33] Baillargeon JP, Iuorno MJ, Jakubowicz DJ, Apridonidze T, He N, Nestler JE (2004). Metformin therapy increases insulin-stimulated release of D-chiro-inositol-containing inositolphosphoglycan mediator in women with polycystic ovary syndrome. J Clin Endocrinol Metab.

