# Expression of Concern to: Does ovary need D-chiro-inositol?

**DOI:** 10.1186/s13048-018-0431-y

**Published:** 2018-07-05

**Authors:** Rosalbino Isabella, Emanuela Raffone

**Affiliations:** 1 C.I.S. Reproductive Medicine, Lamezia Terme, Italy; 2 Obstetrics and Gynecology Department, G. Martino Hospital, Messina, Italy

Pursuant to a decree of the Tribunal of Catania, First Civil Section, LJ Pharma SRL obtained an ex Parte order. The Judge orders BioMed Central Ltd, in the person of the legal representative pro tempore to implement the remedy of the retraction notice on the study, signed by Isabella and Raffone, “Does Ovary need d-chiro-inositol?”, published in 2012 in the specialized journal *Journal* of Ovarian Research [1].
