# Expression of Concern to: Does ovary need D-chiro-inositol?

**DOI:** 10.1186/s13048-018-0470-4

**Published:** 2018-11-23

**Authors:** Rosalbino Isabella, Emanuela Raffone

**Affiliations:** 1 C.I.S. Reproductive Medicine, Lamezia Terme, Italy; 2 Obstetrics and Gynecology Department, G. Martino Hospital, Messina, Italy

A previous Expression of Concern has been published for this article [1]. This is the current Expression of Concern and provides an update to replace the previous Expression of Concern [2].

The Editors of this journal have published this Expression of Concern to alert readers that doubts, that appear to be founded, have been raised about whether proper and effective approval was obtained from an appropriate and independent ethics committee to conduct the clinical study it reports.

Concerns, that appear to be founded, have also been expressed about the scientific content of the article. There has been no response from any of the authors to the Editor’s efforts to seek confirmation about the ethics approval status of this study. Furthermore, in a judicial declaration, Dr. Emanuela Raffone has denied authorship of this article. Pending further investigation, appropriate editorial action might be taken, if additional information becomes available.
