# Supplementary figures and images for: CONCERN: Does ovary need D-chiro-inositol?
Source: J Ovarian Res. 2012 May 15;5:14. doi: 10.1186/1757-2215-5-14 (PMC3447676; doi:10.1186/1757-2215-5-14)

No. Immature oocyte

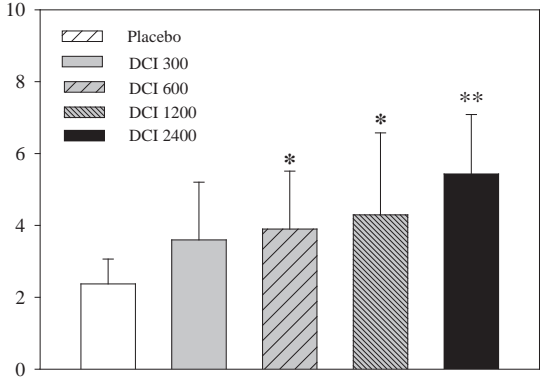

Supplement: Supplementary file 1 — Authors’ original file for figure 1 [file 13048_2012_98_MOESM1_ESM.pdf]

No. MII oocytes

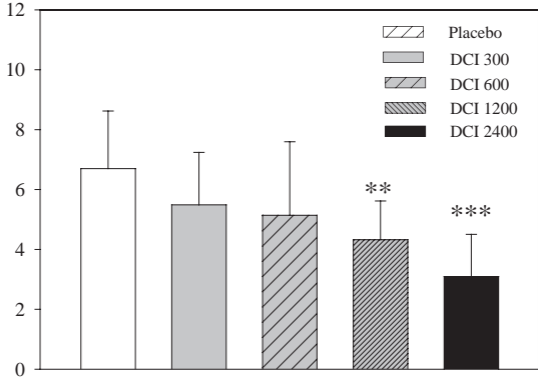

Supplement: Supplementary file 2 — Authors’ original file for figure 2 [file 13048_2012_98_MOESM2_ESM.pdf]

# Embryo Grade I

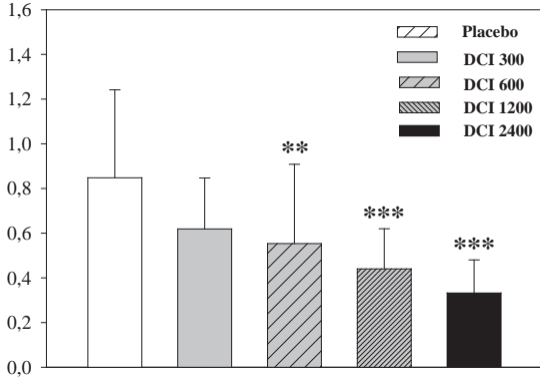

Supplement: Supplementary file 3 — Authors’ original file for figure 3 [file 13048_2012_98_MOESM3_ESM.pdf]
